# Deciphering the function of the CNGB1b subunit in olfactory CNG channels

**DOI:** 10.1038/srep29378

**Published:** 2016-07-11

**Authors:** Vasilica Nache, Nisa Wongsamitkul, Jana Kusch, Thomas Zimmer, Frank Schwede, Klaus Benndorf

**Affiliations:** 1 Institute of Physiology II, Jena University Hospital, Friedrich Schiller University Jena, D-07743 Jena, Germany; 2 BIOLOG Life Science Institute, Flughafendamm 9A, D-28199 Bremen, Germany

## Abstract

Olfactory cyclic nucleotide-gated (CNG) ion channels are key players in the signal transduction cascade of olfactory sensory neurons. The second messengers cAMP and cGMP directly activate these channels, generating a depolarizing receptor potential. Olfactory CNG channels are composed of two CNGA2 subunits and two modulatory subunits, CNGA4, and CNGB1b. So far the exact role of the modulatory subunits for channel activation is not fully understood. By measuring ligand binding and channel activation simultaneously, we show that in functional heterotetrameric channels not only the CNGA2 subunits and the CNGA4 subunit but also the CNGB1b subunit binds cyclic nucleotides and, moreover, also alone translates this signal to open the pore. In addition, we show that the CNGB1b subunit is the most sensitive subunit in a heterotetrameric channel to cyclic nucleotides and that it accelerates deactivation to a similar extent as does the CNGA4 subunit. In conclusion, the CNGB1b subunit participates in ligand-gated activation of olfactory CNG channels and, particularly, contributes to rapid termination of odorant signal in an olfactory sensory neuron.

Cyclic nucleotide-gated (CNG) channels are nonselective cation channels that play an important role for the sensory signaling in the vertebrate visual and olfactory system^1^. These channels are activated by the binding of cyclic nucleotides (cAMP or cGMP)^2^,^3^. Native CNG channels are heterotetramers composed of two or three different types of subunits: 3 x CNGA1 and 1 x CNGB1a in rod channels^4^,^5^, 2 x CNGA3 and 2 x CNGB3 in cone channels^6^, 2 x CNGA2, 1 x CNGA4 and 1 x CNGB1b in olfactory channels^7^,^8^. All subunits share a similar architecture, containing six transmembrane domains (S1 to S6), a pore region between S5 and S6 and an intracellulary located N- and C-terminus. At the C-terminus each subunit contains a cyclic nucleotide-binding domain (CNBD)^2^. Despite these structural similarities, only CNGA1, CNGA2 and CNGA3 subunits are able to form functional channels on their own when expressed heterologously, whereas the CNGB and CNGA4 subunits do not^9^,^10^,^11^. These subunits were therefore considered to be modulatory, and knowledge about the type of modulation has been growing over the last years.

For the heterotetrameric olfactory CNG channels it is known for long that the presence of both the CNGB1b and CNGA4 subunit leads to an increased apparent affinity for cyclic nucleotides^7^,^8^,^10^,^12^,^13^ and accounts for the fast inhibition of native channels by Ca^2+^/CaM^14^,^15^,^16^. In particular the CNGB1b subunit was shown to be critical for Ca^2+^/CaM-mediated desensitization^17^. Moreover, the CNGB1b subunit is also responsible for the ciliary trafficking of the native heterotetrameric channel^18^.

Although all three types of subunits in olfactory CNG channels have a binding domain, it is still elusive whether or not all of them indeed bind a ligand in the heterotetrameric context and, if so, to what extent they actively contribute to channel activation. Studying ligand binding to CNGA4 and CNGB1b subunits has proven difficult because these subunits can only form functional channels when coexpressed with the CNGA2 subunit. By means of functional studies in homotetrameric chimeric channels composed of CNGA2 subunits with the CNBD of CNGA4 (ROON-S2 chimera)^19^,^20^, it was previously demonstrated that the CNGA4 binding domain can bind cAMP and evokes in the CNGA2 background the opening of the pore^21^. Using confocal patch-clamp fluorometry we recently showed, that the native CNGA4 subunit as part of heterotetrameric CNGA2:A4 channels binds a fluorescent cGMP derivative (fcGMP) and that this binding event alone suffices to activate the channel^13^.

In contrast, the contribution of the CNGB1b subunit to channel activation is still unclear, in particular whether or not cyclic nucleotides bind to the CNBD in this subunit at all. When coexpressing the CNGB1b subunit with CNGA2 subunits with disabled binding domains, we did not observe fcGMP binding to the only functional binding site of the CNGB1b subunit in the channel though the CNGB1b subunits were undoubtedly included in the channels^13^. The idea that the CNBD of CNGB1b is not involved in ligand binding is *a priori* not absurd because hERG channels, which play an important role in the repolarization phase of the cardiac action potential^22^, are not regulated by cyclic nucleotides although they contain a structurally related cyclic nucleotide-binding homology domain at their C-terminus^23^,^24^.

In the context of the present work it is noteworthy that the C-linker in CNGA4 prevents the formation of functional CNGA4 homotetramers and that the replacement of a big part of this C-linker with the corresponding residues of CNGA1 is sufficient to generate functional homotetrameric CNGA4 channels^25^. In the line of this one can speculate that a similar mechanism exists also for the CNGB1b subunit, i.e. ligand binding to the CNGB1b subunit could be prevented in the absence of the other types of neighboring subunits. Because in our previous studies on ligand binding to the CNGB1b subunit we expressed either the CNGB1b alone or investigated its properties in CNGA2:B1b channels, but not in heterotetrameric CNGA2:A4:B1b channels, the functional role of the CNGB1b subunit in the activation process is still unknown.

Herein we studied the binding of cyclic nucleotides to the individual subunits and the evoked activation by this binding in heterotetrameric CNGA2:A4:B1b channels with different combinations of selectively disabled binding domains. Our data demonstrate that not only the CNGA4 but also the CNGB1b subunit translates ligand binding to an opening of the channel pore.

## Results

**Figure 1:**
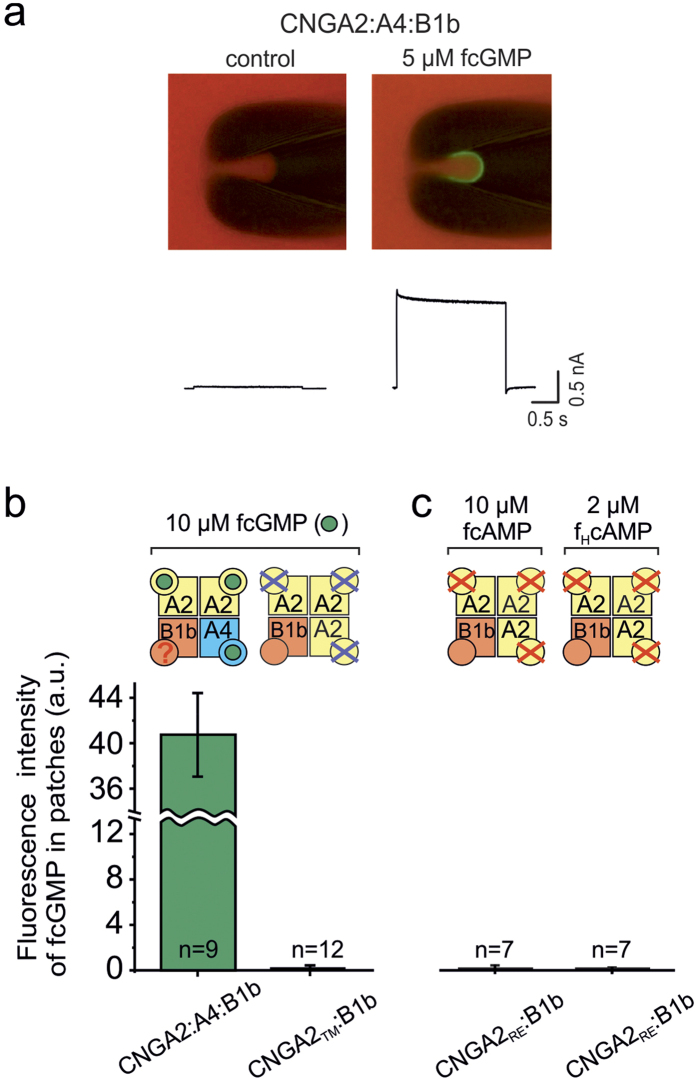
The CNGB1b subunit does not bind cyclic nucleotides when coexpressed with disabled CNGA2 subunits. **(a)** Micrographs of a pipette tip with a patch *in situ* containing multiple CNGA2:A4:B1b channels either in the absence or presence of 5 μM fcGMP. **(b)** Binding of fcGMP to CNGA2, CNGA4 and CNGB1b measured with 10 μM fcGMP. When coexpressed with disabled CNGA2 (CNGA2_TM_), CNGB1b does not bind fcGMP. This result obtained in one of our previous studies^13^ was strengthened with four additional measurements. The fluorescence intensity for CNGA2:A4:B1b channels^13^ is displayed to compare it with that of CNGA2_TM_:B1b channels. (**c**) fcAMP and f_H_cAMP do not detectably bind to CNGB1b. The nonspecific fluorescence, measured in patches from water-injected oocytes, has been subtracted. The cartoons symbolize the four subunits (squares), their binding site (big circle) and the respective ligand (small circle). In all the cartoons, the R538E mutation in the binding domain is indicated by a red cross and the T539M mutation by a blue cross.

The channels to be studied were expressed in *Xenopus* oocytes. We first tested the subunit composition in our channels when coexpressing different combinations of subunits by single-channel recordings. For CNGA2:A4, CNGA2:B1b and CNGA2:A4:B1b channels we exclusively obtained the specific and easily distinguishable single-channel activity reported earlier when injecting respective amounts of RNA^7^,^13^. We therefore assumed that the channels formed had always the expected subunit composition. The specific binding of cyclic nucleotides and the activation of the CNG channels were measured in parallel by means of confocal patch-clamp fluorometry in excised patches, using fluorescent cyclic nucleotide derivatives. The method has been described previously^26^,^27^,^28^. A representative experiment with CNGA2:A4:B1b channels and 5 μM fcGMP is shown in Fig. 1a.

### In CNGA2:B1b channels the CNGB1b subunit neither binds cGMP nor cAMP

To study ligand binding to the CNGB1b subunit selectively, the binding domain of the CNGA2 subunits was disabled by either the point mutation R538E (red crosses in the cartoons) or T539M (blue crosses in the cartoons)^13^,^20^. The R538E mutation leads to a pronounced decrease of the apparent affinity of the respective homotetrameric channels CNGA2_RE_, yielding an *EC*_50_ value for cGMP of 606.7 μM which is much above the value in CNGA2 *wt*-channels (*EC*_50_ = 1.74 μM)^13^. The T539M mutation only moderately decreases the apparent affinity for cGMP in homotetrameric CNGA2_TM_ channels (*EC*_50_ = 10.4 μM) but decreases it much more for fcGMP, resulting in an only negligible activation at 10 μM fcGMP^13^. In one of our previous studies we also showed by confocal patch-clamp fluorometry that fcGMP binds to the CNGA4 subunit but not to the CNGB1b subunit when coexpressed with disabled CNGA2 subunits^13^. Herein, this result was reinforced with four additional measurements (Fig. 1b). Because cAMP is the physiological ligand in olfactory neurons, we tested next whether the CNGB1b subunit in CNGA2:B1b channels binds cAMP. To this end, CNGB1b was coexpressed with CNGA2_RE_. In the presence of 10 μM fcAMP^27^, a concentration at which no ligand binding to the CNGA2_RE_ subunits was expected, we observed no ligand binding to the CNGB1b subunit either (Fig. 1c). This indicates either that fcAMP does not bind to CNGB1b at all or that the affinity of CNGB1b for fcAMP is too low to get detected by our system.

Being aware that the sensitivity of olfactory CNG channels for cAMP is lower than that for cGMP^2^, we applied a novel fluorescent cAMP derivative with an eight times higher potency to open CNGA2:A4:B1b channels compared to fcAMP (f_H_cAMP; *EC*_50_ = 0.59 μM; Supplementary Information and Supplementary Fig. S1). Also f_H_cAMP did not detectably bind to the CNGB1b subunit in CNGA2_RE_:B1b channels (Fig. 1c). To exclude the possibility that the absence of binding is the result of poor channel expression, Fig. 1 contains only patches with a very good channel expression, that produced a current >1.2 nA at 4 mM cGMP.

### In CNGA2:A4:B1b channels the CNGB1b subunit binds cGMP

Together, our previous and the above data show that CNGB1b does neither bind fcGMP, fcAMP, nor f_H_cAMP when expressed with mutated CNGA2 subunits. This is in line with the result that the concentration-activation relationship of homotetrameric CNGA2 channels with cGMP does not differ from that of CNGA2:B1b channels^13^ (Table 1). On the other hand, the concentration-activation relationship of CNGA2:A4:B1b channels is shifted to lower concentrations than that of CNGA2:A4 channels, suggesting that CNGB1b promotes channel activation only in the presence of both CNGA2 and CNGA4. So far it remains open, however, whether the effect of the CNGB1b subunit in CNGA2:A4:B1b channels is generated by the binding of a cyclic nucleotide to the subunit or solely by an allosteric effect of the CNGB1b subunit on the other subunits.

To elucidate the operation of the CNGB1b subunit in the concert with the two CNGA2 subunits and the CNGA4 subunit in CNGA2:A4:B1b channels, we used two mutant subunits CNGA4(R430E) (CNGA4_RE_)^13^ and CNGB1b(R657E) (CNGB1b_RE_)^11^,^17^. These mutations are homologue to CNGA2(R538E) and are therefore indicated in the cartoons in an analogue manner by red crosses. The R/E mutation in either CNGA4 or CNGB1b caused a strong reduction of the apparent cAMP affinity of heterotetrameric channels as observed when the respective subunits were absent from the channel^7^,^17^. These mutated subunits were previously successfully used to characterize the ligand binding to the CNGA4 subunit^13^.

**Figure 2:**
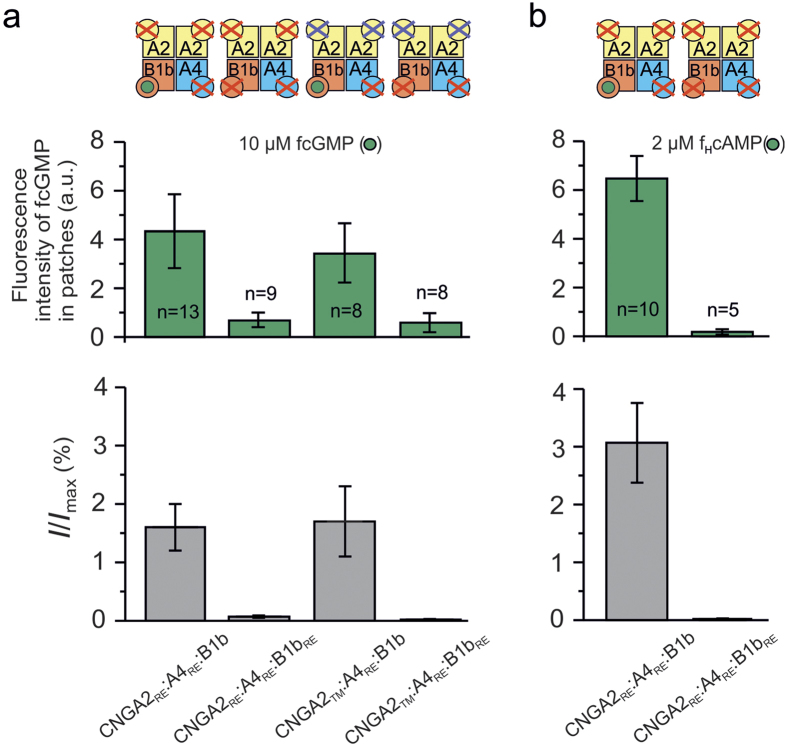
In a CNGA2:A4 background the CNGB1b subunit binds both fcGMP and f_H_cAMP and activates the channel. **(a)** Binding of fcGMP to the CNGB1b subunit evokes channel activation. Binding of fcGMP to CNGB1b was observed when coexpressed with either CNGA2_RE_:A4_RE_ or CNGA2_TM_:A4_RE_ (upper diagram). When disabling the binding domain of CNGB1b, the fluorescence intensity is significantly reduced. Correspondingly, binding to CNGB1b in CNGA2_RE_:A4_RE_:B1b and CNGA2_TM_:A4_RE_:B1b channels evoked moderate channel activation which did not appear when the CNGB1b subunit was disabled (lower diagram). The bar graph shows the current amplitude at 10 μM fcGMP, *I*, corresponding to the binding experiments in the upper diagram, normalized to the current amplitude, *I*_max_, recorded at 4 mM cGMP. **(b)** Binding of f_H_cAMP to CNGB1b was observed when coexpressed with both CNGA2_RE_ and CNGA4_RE_. When disabling the binding domain of CNGB1b, the fluorescence intensity was nearly abolished. Correspondingly, CNGA2_RE_:A4_RE_:B1b channels were activated by the ligand only when the CNGB1b binding site was available.

To verify the hypothesis that the CNGB1b subunit in CNGA2:A4:B1b channels binds cyclic nucleotides, we coexpressed CNGB1b with CNGA4_RE_ and either CNGA2_RE_ or CNGA2_TM_ and measured ligand binding in the presence of 10 μM fcGMP. The result was a robust binding of fcGMP to the CNGB1b subunit (Fig. 2a, upper diagram; Table 2). To verify the specificity of the binding to the CNGB1b subunit in these heterotetrameric channels, we coexpressed the CNGB1b_RE_ subunit with CNGA2_RE_ and CNGA4_RE_. As a result, the binding of fcGMP became essentially diminished, suggesting that the observed fluorescence for CNGA2_RE_:A4_RE_:B1b channels originated predominantly from the binding of fcGMP to CNGB1b. Accordingly, the binding of 10 μM fcGMP to the CNGB1b subunit also triggered activation of both CNGA2_RE_:A4_RE_:B1b and CNGA2_TM_:A4 _RE_:B1b channels to 1.6 ± 0.4% and 1.7 ± 0.6%, respectively, (Fig. 2a, lower diagram) whereas the constructs with all four binding sites disabled, CNGA2_RE_:A4_RE_:B1b_RE_ and CNGA2_TM_:A4_RE_:B1b_RE_, did not trigger activation. Notably, the degree of activation generated by the ligand binding to CNGB1b of 1.6 ± 0.4% and 1.7 ± 0.6% is significantly below that of ~12% induced by the binding of fcGMP to CNGA4 in CNGA2_TM_:A4 channels^13^, suggesting that CNGB1b does contribute to channel activation but only to a small extent. The result that CNGA2_RE_:A4_RE_:B1b_RE_ and CNGA2_TM_:A4_RE_:B1b_RE_ did not produce any current at 10 μM fcGMP suggests that the small amount of fluorescence observed for these constructs originates from non-specific binding.

### In CNGA2:A4:B1b channels the CNGB1b subunit binds cAMP

We next tested whether the CNGB1b subunit in CNGA2_RE_:A4_RE_:B1b channels binds cAMP by applying 2 μM f_H_cAMP as ligand. The result is that similar to fcGMP, the CNGB1b subunit binds f_H_cAMP in the presence of the both CNGA2_RE_ and CNGA4_RE_ (Fig. 2b, upper diagram; see also Table 2). Moreover, f_H_cAMP binding to CNGB1b also triggers channel activation, reaching 3.1 ± 0.7% of the saturating activation measured with 4 mM cGMP (Fig. 2b, lower diagram). Again, binding of f_H_cAMP to the CNGB1b subunit and activation were abolished when also disabling the binding domain in the CNGB1b subunit.

### Apparent affinity of the CNGB1b subunit in CNGA2:A4:B1b channels

Our data so far suggest that all three types of subunits in CNGA2:A4:B1b channels contribute to channel activation by both cGMP and cAMP binding. Among the modulatory subunits, the effect on activation by the ligand binding to the CNGA4 subunit seems to exceed that of the CNGB1b subunit.

In order to determine the apparent affinity of the modulatory subunits in heterotetrameric CNGA2:A4:B1b channels for cGMP, we coexpressed each of them with disabled neighbored subunits, generating themselves activation effects only at much higher ligand concentrations. In case of two well-separated current components, this would allow us to determine the *EC*_50_ value for the CNGB1b and CNGA4 subunit in functional heterotetrameric channels.

**Figure 3:**
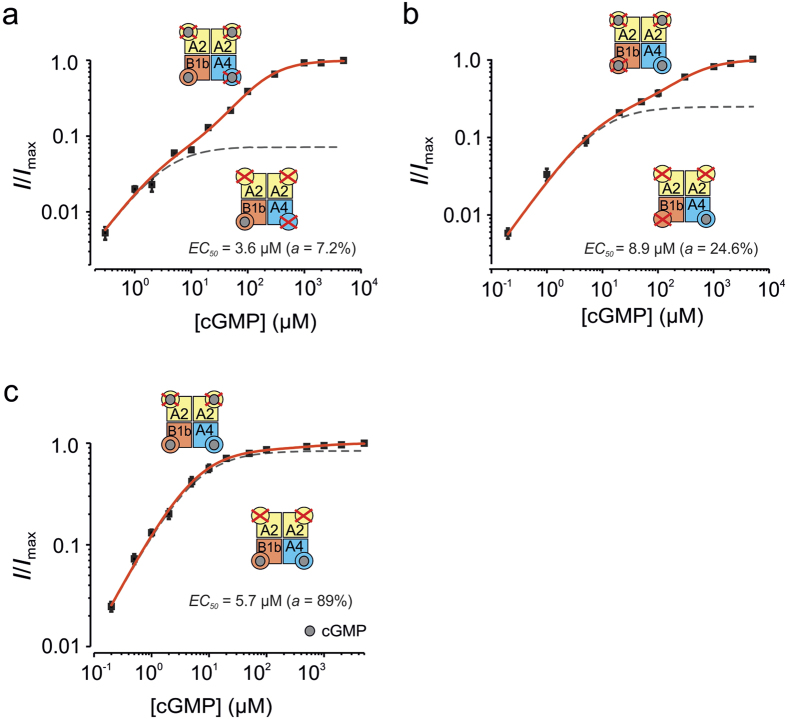
Contribution of the CNGB1b and CNGA4 subunit to channel activation. Concentration-activation relationships for heterotetrameric CNG channels containing different disabled subunits and cGMP. The voltage was +100 mV. The data points represent means of 7–12 measurements each. The continuous curves (red) are the best fit with equation (2). The fits suggest the presence of a component with high apparent affinity (dashed gray lines) and a component with low apparent affinity. **(a)** CNGA2_RE_:A4_RE_:B1b. *EC*_50,l_ = 177.8 μM cGMP, *H*_l_ = 1.2, *EC*_50,h_ = 3.6 μM cGMP, *H*_h_ = 1.0 (fixed), *a* = 0.07. **(b)** CNGA2_RE_:A4:B1b_RE_. *EC*_50,l_ = 364.2 μM cGMP, *H*_l_ = 1.1, *EC*_50,h_ = 8.86 μM cGMP, *H*_h_ = 1.0 (fixed), *a* = 0.24. **(c)** CNGA2_RE_:A4:B1b. *EC*_50,l_ = 733.2 μM cGMP, *H*_l_ = 1.2, *EC*_50,h_ = 5.7 μM cGMP, *H*_h_ = 1.05, *a* = 0.89. The fit parameters are summarized in Table 1.

In both combinations, CNGA2_RE_:A4_RE_:B1b and CNGA2_RE_:A4:B1b_RE_, the concentration-activation relationships were composed of a component of low apparent affinity, presumably caused by the subunits with disabled binding sites, and a component of high apparent affinity, presumably caused by either CNGB1b (Fig. 3a) or CNGA4 (Fig. 3b). Fit of the data points with equation (2) yielded for the action of the CNGB1b subunit *EC*_50,h_ = 3.6 μM cGMP (Fig. 3a) and for the action of the CNGA4 subunit *EC*_50,h_ = 8.9 μM cGMP (Fig. 3b). For the component of low apparent affinity the values were *EC*_50,l_ = 177.8 μM cGMP (Fig. 3a) and *EC*_50,l_ = 364.2 μM cGMP (Fig. 3b), characterizing the combined action of CNGA2_RE_:CNGA4_RE_ and CNGA2_RE_:CNGB1b_RE_, respectively. This suggests a higher apparent affinity for the less efficient CNGB1b subunit in comparison to the CNGA4 subunit. The herein determined *EC*_50,h_ value for CNGA4 of 8.9 μM cGMP is similar to that obtained previously in the presence of CNGA2_TM_ only (6.8 μM fcGMP)^13^, confirming the idea that in CNGA2_RE_:A4:B1b_RE_ channels the CNGB1b_RE_ subunit does not bind but exerts only a minor allosteric effect on the CNGA4 subunit. Another result is noteworthy; the relative contribution of the modulatory subunits to channel activation differs considerably: The maximum contribution of CNGB1b to channel activation is only 7.2% (Fig. 3a) whereas that of CNGA4 reaches 24.6% of the maximum channel activity generated by all subunits (Fig. 3b).

In order to consider the combined apparent affinity of CNGA4 and CNGB1b, we measured the concentration-response relationship of CNGA2_RE_:A4:B1b channels (Fig. 3c; Table 1). Fit of the data points with equation (2) yielded for the component of high apparent affinity *EC*_50,h_ = 5.7 μM cGMP. This value is in-between the ones obtained for CNGA4 and CNGB1b. The major effect of the interaction of CNGA4 and CNGB1b is to enhance the contribution of the high-affinity component to 89% which is much higher than the contribution of each individual subunit.

### Both the CNGA4 and the CNGB1b subunit speed up channel activation and deactivation

**Figure 4:**
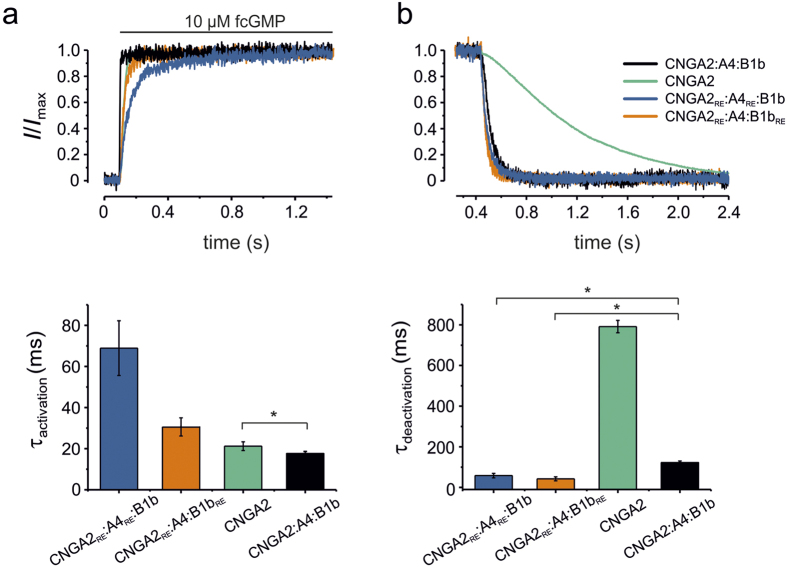
Effects of the CNGB1b and CNGA4 subunit on the kinetics of channel activation and deactivation. Superimposition of activation **(a)** and deactivation **(b)** time courses following a concentration jump to 10 μM fcGMP and back to zero for CNGA2 (green), CNGA2:A4:B1b (black), CNGA2_RE_:A4_RE_:B1b (blue), and CNGA2_RE_:A4:B1b_RE_ (orange) channels. The traces are averages obtained from 5–7 traces obtained from different patches. All traces were normalized with respect to the initial and late current (*I*/*I*_max_). The voltage was set to +10 mV. The respective activation and deactivation time courses of the individual measurements were fitted with monoexponential functions apart from the activation of CNGA2_RE_:A4_RE_:B1b channels that required a biexponential function (Equation S1, Supplementary Information). The time constants are illustrated by bar graphs below the traces. For small differences a *t*-test with respect to CNGA2:A4:B1b channels was performed. An asterisk indicates a significant difference (*p* < 0.05).

To characterize the contribution of each subunit to the kinetics of channel gating, we also studied activation and deactivation time courses for CNGA2:A4:B1b, CNGA2, CNGA2_RE_:A4_RE_:B1b and CNGA2_RE_:A4:B1b _RE_ channels. Fast concentration jumps from zero to 10 μM cGMP and back to zero were applied with the help of a piezo-driven switch pipette^29^. Activation (Fig. 4a) and deactivation (Fig. 4b) time courses were fitted with a single exponential or the sum of two exponential functions in case of the CNGA2_RE_:A4_RE_:B1b channel. The obtained time constants are shown in bar graphs below the traces. In case of the biexponential time courses a mean time constant was obtained according to equation (S1) (Supplementary Information). CNGA2_RE_:A4_RE_:B1b channel activation triggered by CNGB1b only is the slowest, followed by that of CNGA4 only in CNGA2_RE_:A4:B1b_RE_ channels and homotetrameric CNGA2 channels. The most rapid activation was obtained with heterotetrameric CNGA2:A4:B1b channels, suggesting that the accelerating effect of the two modulatory subunits is additive.

In contrast, the accelerating effect of the combination of both modulatory subunits on the deactivation kinetics was much stronger compared to homotetrameric CNGA2 channels. Notably, each of the modulatory subunits alone caused an even faster deactivation than the combination of both. This leads to the conclusion that the modulatory subunits are key players for a rapid channel closure. In olfactory sensory neurons they are therefore proposed to efficiently cease sensory signals.

## Discussion

In this study we demonstrate that in heterotetrameric olfactory CNG channels, composed of two CNGA2 subunits, one CNGA4 subunit and one CNGB1b subunit^2^, not only the CNGA4 subunit but also the CNGB1b subunit actively contributes to channel activation by the binding of a cyclic nucleotide. Moreover, we show that the binding to each of the modulatory subunits alone suffices to trigger partial channel activation without an active support from the CNGA2 subunits (Figs 1, 2, 3). Notably, the active contribution of the CNGB1b subunit requires not only the presence of the CNGA2 subunits but also that of the CNGA4 subunit (Fig. 2). Finally, we show that both the CNGB1b and the CNGA4 subunit markedly accelerate the speed of deactivation and slightly the speed of activation, both with respect to homotetrameric CNGA2 channels (Fig. 4). Methodically we exploited the power of confocal patch-clamp fluorometry to measure ligand binding and activation in parallel and to selectively disable binding sites in the subunits by mutagenesis. Hence, the binding to the modulatory subunits could be tested in heterotetrameric channels in which the other three subunits had disabled binding domains and all allosteric interactions of the subunits were presumably kept.

### Apparent affinity of the subunits in CNGA2:A4:B1b channels

The fact of a significant binding of cyclic nucleotides to both modulatory subunits was strongly supported by the result that disabling all binding domains in heterotetrameric channels notably decreased the fluorescence signal and abolished the current compared to channels in which either a CNGA4 or a CNGB1b *wt*-subunit was present (Fig. 2). When considering the contribution of the subunits to channel activation, our results suggest that the contribution of the CNGB1b subunit is only subordinate in comparison to that of the other modulatory subunit, CNGA4. Extrapolations of the high affinity components in concentration-response relationships yielded a “theoretical” maximum efficiency for the binding of the ligand to the CNGB1b and CNGA4 subunit of ~7% and ~24%, respectively (Fig. 3a,b). In contrast to the efficiency, the apparent affinity, i.e. the potency, for the binding of cGMP to the CNGB1b subunit (*EC*_50_ = 3.6 μM cGMP; Fig. 3a) is higher than that for the binding to the CNGA4 subunit (*EC*_50_ = 8.9 μM cGMP; Fig. 3b). Notably, both these apparent affinities are higher than that of one CNGA2 subunit (EC_50_ = 22.2 μM cGMP) in concatameric homotetrameric CNGA2 channels with three disabled subunits^30^. It will have to be shown by future experiments whether this value holds also for a single CNGA2 subunit in heterotetrameric CNGA2:A4:B1b channels.

When coexpressing CNGB1b and CNGA4 with disabled CNGA2 subunits, the combined contribution of the two modulatory subunits to channel activation reached a much higher efficiency of 89% and the component of high apparent affinity describing the action of both subunits had an *EC*_50_ = 5.7 μM cGMP, a value in-between the values obtained for the individual modulatory subunits (Fig. 3c). Based on these results we propose for cGMP that the CNGB1b subunit plays the role of a “sensor” subunit, to trigger early channel reaction on a sudden increase of the ligand concentration but to exert only a minor effect on channel activation.

Regarding the order of ligand binding to the different subunits it has been suggested that CNGA4 is the first ligand binding subunit at low cAMP concentrations, and the channel can only be opened with the help of CNGA2 upon binding of the second ligand^17^. This proposal was based on functional studies and on the well-established effect of CNGA4 to generate higher ligand sensitivity in heterotetrameric CNGA2:A4:B1b than in homotetrameric CNGA2 channels^31^. Our data with cGMP as ligand deviate from this proposal: In heterotetrameric CNGA2:A4:B1b channels, the CNGB1b subunit alone develops high ligand sensitivity and modest activation (Fig. 3a) and the CNGA4 subunit alone develops some lower ligand sensitivity but noticeably more activation (Fig. 3b).

### Gating kinetics

The results of our study also show that the CNGA4 and CNGB1b subunit together slightly accelerate channel opening (Fig. 4a). By comparing activation kinetics of channels with four functional subunits (CNGA2:A4:B1b and CNGA2) and channels with only one functional modulatory subunit (CNGA2_RE_:A4_RE_:B1b and CNGA2_RE_:A4:B1b _RE_), we showed that both modulatory subunits are necessary to accelerate activation with respect to CNGA2 channels whereas activation induced by the ligand binding to CNGB1b or CNGA4 alone was slower than that in CNGA2 channels.

When comparing deactivation kinetics of the same channels the situation differs substantially: homotetrameric CNGA2 channels deactivate with a much slower time course than channels that are operated by a functional CNGB1b or CNGA4 subunit only (Fig. 4b). Notably, deactivation is already slower in channels containing both accelerating modulatory subunits *and* two functional CNGA2 subunits. This result suggests that the presence of two functional CNGA2 subunits imparts a slowing effect in the deactivation process of heteromultimeric channels. Despite the complexity of the effects by the modulatory subunits on channel kinetics, our results suggest that both of them accelerate the onset and the termination of a sensory signal, resulting in CNGA2:A4:B1b channels in an activation time constant of ~17 ms and a deactivation time constant of ~120 ms. This acceleration should be of relevance for a rapid response of the olfactory system. Our data correlate well with previously reported results regarding the odorant detection which takes aproximately150–250 ms after the inhalation begins^32^,^33^. Thus, the modulatory subunits would enable the OSNs to respond rapidly to a stimulus and, with a much stronger effect, to stop the response of the neuron after an odor molecule has dissociated from its receptor. Rapid termination of the sensory signal would enable OSNs to recover for a subsequent stimulation^33^.

### Outlook

Concerning the result that binding to the CNGB1b subunit is only possible when also the other two types of subunits are present indicates that the CNGB1b subunit exerts its function only when the channels are composed correctly. It has already been shown by several groups that the subunit composition is critical for channel function, influencing binding affinity and trafficking of the channels to the plasma membrane^18^,^34^. Moreover, the CNGB1b subunit has been proposed to be the most critical subunit for the correct channel trafficking, suggesting that olfactory sensory neurons have a complex quality control mechanism which prevents incompletely assembled channels from reaching the ciliary plasma membrane^34^,^35^,^36^ and after arrival, to modulate the channel function.

Our results are potentially of relevance also for other CNG channels. For rod CNGB1a and cone CNGB3 modulatory subunits it has already been shown that they cannot form functional channels in the absence of CNGA1 and CNGA3, respectively, and that they are also critical for channel trafficking^37^,^38^. Until now experimental evidence for ligand binding to CNGB1a and CNGB3 is still missing. Based on our results it is intriguing to speculate that these modulatory subunits play analogue roles in their channels as shown herein for the CNGB1b subunit and eventually also the CNGA4 subunit.

## Methods

### Molecular Biology

Rat olfactory channel subunits CNGA2 (accession No. AF126808), CNGA4 (accession No. U12623), and CNGB1b (accession No. AF068572), as well as all genetically modified subunit variants were subcloned in front of the T7 promoter of pGEMHEnew^39^. Binding sites for cyclic nucleotides were modified by site-directed mutagenesis using a recombinant PCR technique. Correctness of the sequences was confirmed by restriction analysis and DNA sequencing. Preparation of cRNA was done using the mMESSAGE mMACHINE T7 Kit (Ambion, Austin, TX).

### Preparation of oocytes and cRNA injection

Oocytes were harvested surgically under anesthesia (0.3% 3-aminobenzoic acid ethyl ester) from adult females of *Xenopus laevis*. The procedures had approval from the authorized animal ethical committee of the Friedrich Schiller University Jena. The methods were carried out in accordance with the approved guidelines.

The oocytes were treated with collagenase A (3 mg/ml; Roche, Grenzach-Wyhlen, Germany) for 105 min in Ca^2+^-free Barth’s solution containing (in mM) 82.5 NaCl, 2 KCl, 1 MgCl 2, and 5 Hepes, pH 7.5. Stages IV and V oocytes were manually dissected and injected with ~50 ng of cRNA encoding rat olfactory CNGA2:A4:B1b channels (2:1:1 cRNA injection ratio)^8^. The injected oocytes were placed at 18 °C for up to 6 days in Barth’s solution containing (in mM) 84 NaCl, 1 KCl, 2.4 NaHCO_3_, 0.82 MgSO_4_, 0.41 CaCl_2_, 0.33 Ca(NO_3_)_2_, 7.5 TRIS, cefuroxime (4.0 μg × ml^−1^), and penicillin/streptomycin (100 μg × ml^−1^), pH 7.4.

### Chemicals

cGMP and cAMP were obtained from Sigma.

8-[DY-547]-AET-cGMP (fcGMP) and 8-[DY-547]-AET-cAMP (fcAMP) was prepared as reported previously^26^,^27^. The synthesis of 8-[DY-547]-AHT-cAMP (f_H_cAMP) is described in Supplementary Information.

### Current recording

Patch-clamp experiments were performed using an Axopatch 200B amplifier (Molecular Devices LLC, CA, U.S.A.) in the inside-out configuration. The patch pipettes for recording of macroscopic currents were pulled from borosilicate glass tubing (outer diameter 2.0 mm, inner diameter 1.0 mm; Hilgenberg GmbH, Germany). The initial resistance was 0.7–1.3 MΩ. The patches were formed as pushed inside-out patches^40^. The experimental solutions with different concentrations of cGMP, cAMP, 8-[DY-547]-AET-cGMP (fcGMP), 8-[DY-547]-AET-cAMP (fcAMP) or 8-[DY-547]-AHT-cAMP (f_H_cAMP) were administered via a multi-barrel application system to the cytosolic face of the patch. Cyclic nucleotide concentration jumps were performed using a double-barreled glass pipette mounted on a piezo-driven device. Intracellular (bath) and extracellular (pipette) solutions contained (mM) 150 KCl, 1 EGTA, 5 Hepes, pH = 7.4 with KOH. Each patch was initially exposed to a solution containing no cyclic nucleotide and then to a solution containing the saturating concentration of either 100 μM cGMP, 4 mM cGMP or 1 mM cAMP. Currents in the absence of the cyclic nucleotide were subtracted. All patches included in this study were first tested for channel expression in the presence of a saturating ligand concentration. Only patches with large current, i.e. a large number of channels, were included in our statistics. The currents were measured at +10 mV or, if mentioned, at +100 mV.

### Confocal patch-clamp fluorometry

The ligand binding was measured in macropatches by patch-clamp fluorometry^41^,^42^ combined with confocal microscopy^26^,^27^,^43^. By means of fluorescently-labeled cGMP and cAMP derivatives, this technique allowed us to simultaneously measure ligand binding and gating in olfactory CNG channels. Recordings were performed with an LSM 510 or 710 confocal microscope (Zeiss, Jena, Germany) and were triggered by the ISO3 software (MFK, Niedernhausen, Germany). To distinguish between the fluorescence of the unbound ligands from that of the bound ligands, a second dye, DY647 (Dyomics, Jena, Germany), was added to the bath solution. The 543-nm and 633-nm lines of a He-Ne laser were used to excite fcAMP/fcGMP and DY647, respectively. The method of confocal patch-clamp fluorometry has been described in detail previously^26^.

### Data Acquisition and Analysis

Measurements were performed with the ISO2 and ISO3 soft- and hardware (MFK Niedernhausen, Germany). The sampling rate was 5 kHz, the filter was set to 1 kHz.

Fits of equations to data points were performed with the 8.1G^®^ software (OriginLab Corporation, Northampton, U.S.A.). Concentration-activation relationships were obtained by relating the actual current amplitude, *I*, to the maximum current amplitude elicited by a saturating concentration of ligand, *I*_max_, and plotting this ratio as function of the ligand concentration, *x*. The concentration-activation data were fitted using the Hill equation


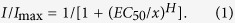


where *EC*_50_ is the ligand concentration of half maximum effect and *H* the Hill coefficient.

For the concentration-activation relationships showing two components, the data points were fitted to the sum of two Hill equations according to


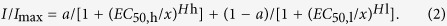


*EC*_50,h_, *EC*_50,l_, *H*_h_, and *H*_l_ are the ligand concentration of half maximum current and the Hill coefficient for the high and low affinity component, respectively. *a* and (1-*a*) denote the contribution of the high and low affinity component, respectively.

Statistical data are given as mean ± s.e.m.

## Additional Information

**How to cite this article**: Nache, V. *et al*. Deciphering the function of the CNGB1b subunit in olfactory CNG channels. *Sci. Rep.* **6**, 29378; doi: 10.1038/srep29378 (2016).

## Supplementary Material

Supplementary Information

## Figures and Tables

**Table 1:** Apparent affinity of CNG channels containing different subunit composition.

Constructs	cGMP (μM)					
	*EC*_50_	*H*	*EC*_50,l_	*H*_1_	*EC*_50,h_	*H*_h_
CNGA2	1.74	1.9				
CNGA2_RE_*	606.7	2.7				
CNGA2_TM_*	10.4	2.3				
CNGA2:A4	1.28	1.7				
CNGA2:B1b	1.9	2.0				
CNGA2:A4:B1b	0.76	2.0				
CNGA2_RE_:A4_RE_:B1b			177.8	1.2	3.6	1**
CNGA2_RE_:A4:B1b _RE_			364.2	1.1	8.86	1**
CNGA2_RE_:A4:B1b			733.2	1.2	5.7	1.1

^*^from Nache *et al*., 2012.

^**^H was set to unity.

Fit parameters for concentration-activation relationships of CNG channels containing different compositions of subunits with either functional or disabled binding domains. The relationships for the constructs 1 to 6 were fitted with Equation 1 and for the constructs 7 to 9 with Equation 2.

**Table 2:** Ligand binding to the modulatory subunits.

Constructs	CNGA4	CNGB1b		
	fcGMP	fcGMP	fcAMP	f_H_cAMP
	10 μM	10 μM	10 μM	2 μM
CNGA4*	−			
CNGB1b*		−		
CNGA2_RE_:A4*	+			
CNGA2_TM_:A4	+			
CNGA2_TM_:A4_RE_*	−			
CNGA2_RE_:B1b		−	−	−
CNGA2_TM_:B1b		−		
CNGA2_RE_:A4_RE_:B1b	−	+		+
CNGA2_RE_:A4_RE_:B1b_RE_	−	−		−
CNGA2_TM_:A4_RE_:B1b	−	+		
CNGA2_TM_:A4_RE_:B1b_RE_	−	−		

^*^from Nache *et al*., 2012.

The symbols “+” and “−” indicate that ligand binding to the respective modulatory subunits was either observed or not.
