# Corrigendum: Deciphering the function of the CNGB1b subunit in olfactory CNG channels

**DOI:** 10.1038/srep47000

**Published:** 2018-06-28

**Authors:** Vasilica Nache, Nisa Wongsamitkul, Jana Kusch, Thomas Zimmer, Frank Schwede, Klaus Benndorf

Scientific Reports 6; Article number: 2937810.1038/srep29378; published online: 07 11 2016; updated: 06 28 2018

The Acknowledgements section in this Article is incomplete.

“We like to thank to K. Schoknecht, S. Bernhardt, and A. Kolchmeier for excellent technical assistance. This work was supported by grants of the Deutsche Forschungsgemeinschaft to K.B. and V.N. and of the Friedrich Schiller University to V.N.”

should read:

“We like to thank to K. Schoknecht, S. Bernhardt, and A. Kolchmeier for excellent technical assistance. This work was supported by the Deutsche Forschungsgemeinschaft (TRR 166 ReceptorLight; project A5 to K.B; project B1 to V.N.) and by the Friedrich Schiller University to V.N.”

