# Supplementary material for: Deciphering the function of the CNGB1b subunit in olfactory CNG channels
Source: Sci Rep. 2016 Jul 11;6:29378. doi: 10.1038/srep29378 (PMC4942689; doi:10.1038/srep29378)
Supplement: Supplementary Information [file srep29378-s1.pdf]

Supplementary Information

to the manuscript

Deciphering the function of the CNGB1b subunit in olfactory CNG channels

by Vasilica Nache*, Nisa Wongsamitkul, Jana Kusch, Thomas Zimmer, Frank Schwede, Klaus Benndorf*

*To whom correspondence should be addressed:

Vasilica.Nache@med.uni-jena.de or Klaus.Benndorf@med.uni-jena.de

SUPPLEMENTARY METHODS

Synthesis of 8-[DY-547]-AHT-cAMP

General Synthetic Methods. All reagents were of analytical grade, HPLC grade or better available from commercial suppliers. Dimethyl sulfoxide (DMSO) was stored over activated molecular sieves for at least two weeks before use. UV spectra were recorded with a V-650 spectrophotometer (JASCO, Groß-Umstadt, Germany). Mass spectra were generated with an Esquire LC 6000 spectrometer (Bruker Daltronics, Bremen, Germany) in the ESI-MS mode with 50 % water / 50 % methanol as matrix.

All analytical reversed phase HPLC analyses were performed at 30°C and were accomplished with a LaChrom Elite instrument consisting of a L-2130 pump, a L-2455 diode array detector, a L-2350 column oven, and EZChrom software version 3.3.1 SP1 (all VWR-Hitachi, Hannover, Germany). Eluent composition was either 18% CH_3CN, 20 mM triethylammonium formate (TEAF), pH 6.8, 1 mL/min or 22% CH_3CN, 20 mM triethylammonium formate (TEAF), pH 6.8, 1 mL/min. The stationary phase was YMC ODS-A 12 nm, S-11 µm (YMC, Dinslaken, Germany) in a 250 x 4.6 mm stainless steel column with a Gemini C18, 4 x 3 mm SecurityGuard column (Phenomenex, Aschaffenburg, Germany). Semipreparative HPLC purification was performed at ambient temperature with a LC-8A pump (Shimadzu, Duisburg, Germany), a preparative K 2001 UV-detector (Knauer, Berlin, Germany) and a L200E analog recorder (Linseis, Selb, Germany). YMC ODS-A 12 nm, S-11 µm (YMC), in a 250 x 16 mm stainless steel column and a C18, 10 x 10 mm SecurityGuard cartridge (Phenomenex, Aschaffenburg, Germany).

Synthesis of 8-(6-[DY-547]-aminohexylthio)adenosine-3',5'-cyclic monophosphate, (8-[DY-547]-AHT-cAMP / f_HcAMP)

8-[DY-547]-AHT-cAMP (f_HcAMP) was synthesized in accordance to reported procedures for

the related 8-[DY-547]-AET-cGMP (fcGMP) (Biskup et al., 2007) and 8-[DY-547]-AET-cAMP (fcAMP) (Kusch et al., 2010) with minor modifications: 17.7 mg (30 µmol) of 8-(6-Aminohexylthio)adenosine-3',5'-cyclic monophosphate (8-AHT-cAMP), *N*,*N*-diisopropylamonnium salt, (BIOLOG LSI, Bremen, Germany) and 8.6 µl (50.2 µmol) *N*,*N*-diisopropylethylamine were dissolved in 4000 µl absolute DMSO and divided into two 3.5 mL polypropylene vials with screw cap. To each of these vials 5 mg (6.8 µmol) DY-547-NHS ester (Dyomics, Jena, Germany), pre-dissolved in 200 µL absolute DMSO, was added. The resulting mixtures were placed in a MHL 20 thermomixer (HLC Biotech, Bovenden, Germany) set at 25°C and shaken at 950 rpm for 75 min. The progress of the reactions was monitored by analytical reversed phase HPLC. After completion of the reactions the volume of the raw mixtures was reduced to ~100 µL in a SC100 SpeedVac concentrator (Savant, Farmingdale, NY, U.S.A.), diluted with 7 mL 16 % CH_3CN, 20 mM triethylammonium formate (TEAF), pH 6.8 and purified by semipreparative reversed phase HPLC. The target compound was eluted with 16 - 18% CH_3CN, 20 mM TEAF, pH 6.8, 5 mL/min, as a mixture of diastereomeric isomers (DY-547 is a chiral fluorescent dye). Subsequent cation exchange to sodium and desalting was accomplished by the same semipreparative HPLC set-up. After washing with 100 mM NaH_2PO_4 (pH 6.8) and water, 8-[DY-547]-AHT-cAMP was eluted with a gradient of 0 - 100% methanol and product-containing fractions were evaporated with a rotary evaporator under reduced pressure. 9.9 µmol of 8-[DY-547]-AHT-cAMP, sodium salt, with a purity of 99.46% by HPLC at 280 nm were obtained as isomeric mixture (yield: 72.8%). A separation of the diasteromeric isomers was not possible with our HPLC system. Successful synthesis was confirmed by mass spectrometry. Formulas: for free acid form: $C_{46}H_{59}N_8O_{13}PS_3$ (MW: 1059.2); for disodium salt: $C_{46}H_{57}N_8O_{13}PS_3Na_2$ (MW: 1103.2). Negative mode: m/z of 1057.5 $(M-H)^-$, 1079.5 $(M-2H+Na)^-$. Positive mode: m/z of 1059.6 $(M+H)^+$, 1081.6 $(M+Na)^+$, 1103.5 $(M-H+2Na)^+$.

Fit of the activation and deactivation time courses

The activation and deactivation time courses for the CNGA2:A4:B1b, CNGA2, CNGA2$_{RE}$:A4$_{RE}$:B1b and CNGA2$_{RE}$:A4:B1b$_{RE}$ channels were obtained by performing fast concentration jumps from zero to 10 µM fcGMP and back to zero with the help of a double-barreled glass pipette mounted on a piezo-driven device. The respective time courses were fitted with a single exponential function for all measured constructs with the exception of CNGA2$_{RE}$:A4$_{RE}$:B1b which needed a biexponential function. For the CNGA2$_{RE}$:A4$_{RE}$:B1b a mean time constant, τ_{mean}, was determined according to

$$\tau_{mean} = (A_1 \times \tau_{fast} + A_2 \times \tau_{slow})/(A_1 \times A_2) \qquad \text{(S1)}$$

where τ_{fast} and τ_{slow} are the activation time constants, and A_1 and A_2 are the amplitudes of the respective components. This approach allowed us to compare the activation and deactivation speed for all constructs (Fig. 4, bottom bar graphs).

SUPPLEMENTARY FIGURES

Supplementary Fig. S1. f_HcAMP shifts the concentration-activation relationship of heterotetrameric CNG channels to smaller concentrations.

a

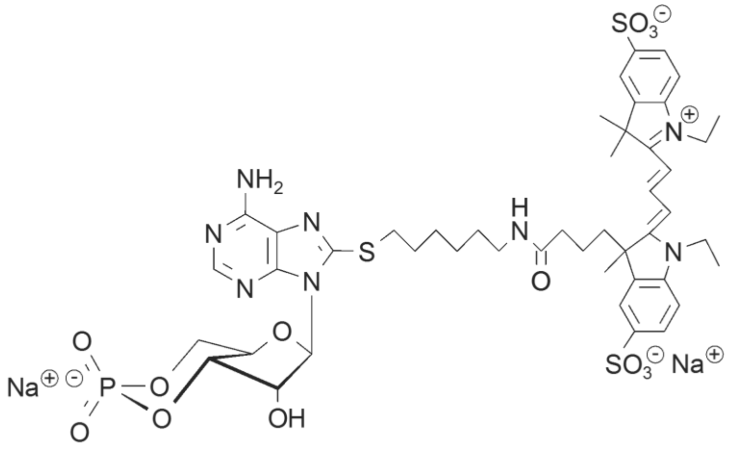

b

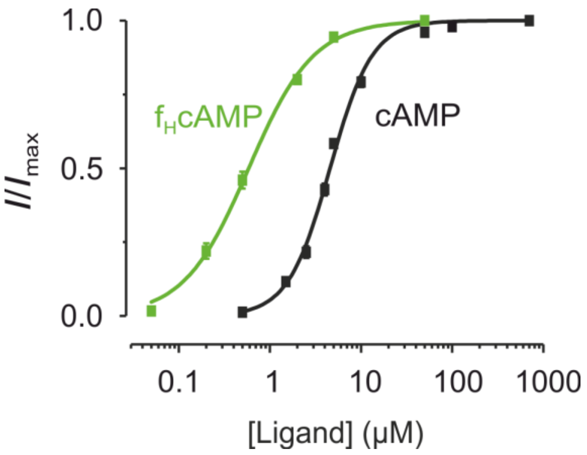

(a) Structure of 8-[DY-547]-AHT-cAMP (f_HcAMP). In f_HcAMP the dye DY547 is coupled to the cAMP moiety by a hexyl linker instead of an ethyl linker in fcAMP.

(b) Concentration-activation relationships of the CNGA2:A4:B1b channels activated by either cAMP (black) or f_HcAMP (green) under steady-state conditions. The currents were recorded at +10 mV. Data points, obtained from 7-10 patches each, were fitted by equation (1) yielding the following parameters: cAMP, EC_{50} = 4.57 µM, H = 1.9; f_HcAMP, EC_{50} = 0.59 µM, H = 1.2.
